# Analysis of Various Laparoscopic Pediatric Urology Surgeries: Five-Year Experience of a Single Institution

**DOI:** 10.7759/cureus.63806

**Published:** 2024-07-04

**Authors:** Sevim Yener, Zekeriya Ilce

**Affiliations:** 1 Department of Pediatric Urology, University of Health Sciences, Umraniye Training and Research Hospital, Istanbul, TUR; 2 Department of Pediatric Surgery, University of Health Sciences, Umraniye Training and Research Hospital, Istanbul, TUR

**Keywords:** anomaly, laparoscopy, kidney, genitourinary, experience

## Abstract

Background

In this study, we aim to report our single-center experience with laparoscopic pediatric urological surgeries. We aim to determine the feasibility of various urological and urogenital laparoscopic procedures and the tricks that increase surgical success.

Methodology

Data from 98 patients who underwent laparoscopic urological and/or urogenital procedures for diagnostic and therapeutic purposes in our clinic between June 2018 and February 2023 were retrospectively analyzed. All surgeries were performed by the same surgical team. Laparoscopic procedures included orchidopexy, gonadectomy, vaginoplasty, hysterectomy, pyeloplasty, nephrectomy/partial nephrectomy, ureteroneocystostomy, bladder diverticulum excision, renal cyst excision, proximal ureter stone removal, oophorectomy, ovarian detorsion, oophoropexy, and lymph node excision for diagnostic purposes. The surgical planning of the patients was based on the decisions of the pediatric nephrology, pediatric endocrinology, and pediatric oncology departments and the multidisciplinary council. Demographic characteristics of the patients, surgical indications, and intraoperative data, as well as postoperative pathological diagnoses and complications, were recorded. All patients underwent a transperitoneal approach. The duration of the operation was obtained from anesthesia records and defined as the time from the beginning of the surgical incision to the closure of the skin incision.

Results

Of the patients, 54 were males and 44 were females. The median age was 7.8 years. No complications other than grade 1 according to the Clavien-Dindo classification were observed in our patients. As different types of surgeries were analyzed, the mean operative duration was estimated.

Conclusions

The laparoscopic method should be performed by surgeons experienced in advanced surgeries in pediatric urology. It is critical to consider the difference in the size of pediatric patients in preparation for laparoscopic surgery to minimize technical and ergonomic problems. We believe that each surgery has its specific tricks and that these should be a part of laparoscopy training. Moreover, developing and sharing this information would be very useful for pediatric urologists.

## Introduction

Currently, with the development of laparoscopic surgical equipment and the spread of laparoscopy training, the use of this method in pediatric urological procedures has been increasing. In pediatric urology practice, many open surgical techniques can be performed laparoscopically without compromising approach standards. Laparoscopy has been shown to be associated with less postoperative pain, minor surgical scars, decreased costs, and lower bleeding rates when compared to laparotomy, as well as having certain advantages such as shorter hospital stays [1]. Recognizing anomalies accompanying urinary system pathologies in children prevents serious complications. In cases where the laparoscopic approach is preferred in pediatric patients, the ergonomics of creating a surgical working area and the length of the learning curve should be taken into consideration [2]. When planning a laparoscopic intervention, recognizing the surgical anatomy, variations, and rare anomalies is the appropriate approach. In this study, we present our single-center experience of various laparoscopic surgeries performed in pediatric urology practice. We aim to determine the feasibility of various urological and urogenital laparoscopic procedures and the tricks that increase surgical success.

## Materials and methods

This single-center, retrospective, cross-sectional study included laparoscopic urological and urogenital surgeries performed between June 2018 and February 2023. A total of 98 patients who underwent laparoscopic urological and/or urogenital procedures for diagnostic and therapeutic purposes were included. Patients who underwent open surgery were not included. No comparison was made between the procedures.

The specific purpose of this study is to highlight the merits of a wide variety of laparoscopic procedures that can be performed at a single center. Medical data of the patients were retrieved from the hospital database. Data including demographic characteristics of the patients, surgical indications, and intraoperative data, as well as postoperative pathological diagnoses and complications, were recorded. Duration of operation was obtained from anesthesia records and defined as the time from the beginning of the surgical incision to the closure of the skin incision. The study protocol was approved by the Health Sciences University Umraniye Training and Research Hospital Ethics Committee (approval number: B.10.1.TKH.4.34.H.GP.0.01/124, dated 24.04.2023). The study was conducted in accordance with the principles of the Declaration of Helsinki.

All patients underwent a transperitoneal approach. Surgeries were performed by the same surgical team. Procedures were classified under three headings, namely, urinary system surgeries, urogenital system surgeries, and other surgeries. Due to the diversity of surgical types, we aim to share our experiences to determine the necessity of a patient-based approach for each technique and the situations that should be taken into consideration in surgical techniques to facilitate the surgeon’s work. Comparative results are not planned. Urinary system surgeries included pyeloplasty, nephrectomy/partial nephrectomy, ureteroneocystostomy (UNC), bladder diverticulum excision, renal cyst excision, and proximal ureter stone removal. Urogenital system surgeries included orchidopexy, gonadectomy, vaginoplasty, hysterectomy, oophorectomy, ovarian/tubal detorsion, and oophoropexy. Other surgeries included lymph node excision and diagnostic interventions. In all patients, a Foley catheter was used for bladder decompression to prevent any potential bladder injury during the first trocar insertion. Patients were positioned in the lateral position for surgeries such as nephrectomy, partial nephrectomy, or pyeloplasty and in the supine position for procedures such as undescended testicles, UNC, and diagnostic laparoscopy. All patients underwent laparoscopic port insertion using the open Hasson technique.

Patients’s age at surgery, preoperative preparations (diagnostic or interventional), type of surgery, median operation time, pre and postoperative complications, and days of hospital stay were collected from patient files and analyzed.

Statistical analysis

In our study, simple statistics were used, and the mean ± SD and percentage values of the groups were obtained from the statistical calculation in a Microsoft Excel worksheet (Microsoft Corp., Redmond, WA, USA).

## Results

Of the patients, 54 were males and 44 were females with a median age of 7.8 ± 2.8 years. Laparoscopic surgeries are summarized in Table 1.

**Table 1:** Demographic, intra, and postoperative data of the patients (n = 98).

Operation		Number of operations	Number of patients	Median age, years (range)	Median operative time, minutes (range)	Postoperative complications	Median hospital stay, days (range)
Ectopic testis exploration/Orchiopexy		14/17 (14.2%/17.3%)	31 (31.6%)	37 (10–69)	38 (43–68)	1	1–2
Pyeloplasty		16 (16.3%)	16 (16.3%)	45 (27–65)	101 (123–210)	0	3–5
Ovarian pathologies	Ovarian cyst/Tubal cyst	8/1 (8.1%/1%)	21 (21.4%)	13–17	35–55	0	1–2
	Ovarian torsion/Tubal torsion	5/2 (5.1%/2%)		10–16	30–45	0	1–2
	Ovarian mass excision	5 (5.1%)		14–17	45–60	0	1–5
Partial nephrectomy/Total nephrectomy		6/2 (6.1%/2%)	8 (8.1%)	3–12	125–145	0	2–5
Ureteroneocystostomy		6 (6.1%)	6 (6.1%)	3–6	99–181	0	3–5
Bladder diverticulum excision		3 (3%)	1(1%)	3–6	150–195	0	3–5
Diagnostic		5 (5.1%)	5 (5.1%)	3–16	40–53	0	1–2
Oophoropexy		2 (2%)	2 (2%)	2–3	61–67	0	1–2
Gonadectomy		2 (2%)	2 (2%)	4–5	53–68	0	1–2
Bilateral oophorectomy + hysterectomy		1 (1%)	1 (1%)	16	122	0	2
Vaginoplasty		2 (2%)	2 (2%)	14–17	250–360	0	5–10
Renal cyst excision		1 (1%)	1 (1%)	15	62	0	1
Proximal ureter stone removal		1 (1%)	1 (1%)	13	64	0	2
Lymph node excision		1 (1%)	1 (1%)	15	50	0	1

Patients with hydronephrosis on ultrasonography underwent voiding cystourethrogram. Patients without reflux underwent pyeloplasty after dynamic renal scintigraphy. Preoperative cystoscopy and retrograde pyelography were performed in all patients who underwent laparoscopic pyeloplasty. Of the two patients who underwent total nephrectomy, one had a nonfunctional kidney and the other had a renal mass. Only one of the patients who underwent partial nephrectomy had an incomplete double system anomaly. All others had incomplete double system anomalies. Static renal scintigraphy, complete urinalysis, and urine culture results of patients who underwent surgery for vesicoureteral reflux (VUR) were reported. Before UNC, the history of subureteric injection and the degree of reflux were evaluated. A ureteral stent was placed in patients undergoing UNC via preoperative cystoscopy to facilitate dissection. While the bladder diverticulum was being removed, a preoperative Fogarty catheter was placed into the diverticulum. Kidney cyst excision was performed using a vein sealer to control bleeding and was removed from the umbilicus. The patient with proximal ureter stone and hydronephrosis underwent stone removal and ureter repair via ureterotomy.

Surgery for undescended testicles was determined mainly by the length of the gonadal vessels. The testicle was dissected and adequately mobilized until it reached the contralateral inner ring by gross measurement. A gonadectomy was performed in two patients due to an atrophic gonad located in the abdomen. Bilateral oophorectomy and hysterectomy were performed for the patient with a large-sized ovarian cyst who was being followed up due to congenital adrenal hyperplasia due to the council’s decision to develop in a male gender. A ureteral catheter was placed in two patients who underwent vaginoplasty for ureter safety, and the hematometra fluid was drained with an aspirator advanced to the uterus for ease of dissection. Ovarian detorsion, ovarian-sparing cyst excision, total mass excision for ovarian masses, and contralateral biopsy were performed based on macroscopic findings and frozen section results. The mass was taken out of the abdomen with an endobag without rupturing. Tubal detorsion fixation was performed in two cases with isolated tubal torsion. The ovaries of two patients diagnosed with Ewing sarcoma and rhabdomyosarcoma, for whom pelvic radiotherapy was planned, were fixed to the anterior superior abdominal wall. Lymph node excision was performed with a vessel sealer in the patient diagnosed with familial Mediterranean fever. Diagnostic explorative laparoscopy was performed on five patients.

No complications other than grade 1 according to the Clavien-Dindo classification were observed in our patients.

Laparoscopic pyeloplasty was performed in 16 patients with the diagnosis of ureteropelvic junction stenosis. During laparoscopy, a 5/0 absorbable suspension suture was placed on the proximal ureter. Pelvic reduction was performed in patients with very wide pelvis and a double J stent was placed in all of them. In patients in whom a double J stent could not be placed preoperatively, a 4 F double J stent advanced through a 14 F branule was placed intraoperatively. The anesthesia team recommended intravenous fluid restriction to prevent the urine draining into the abdomen from distorting the camera image during the incision of the pelvis.

Partial heminephrectomy was performed in six patients due to recurrent urinary tract infection and nonfunctional upper pole. Data of the patients are presented in Table 2. One patient underwent laparoscopic right upper pole heminephrectomy at an external center and had recurrent urinary tract infection in the postoperative fifth year. During follow-ups, there was a 5 × 5 cm loculated collection in the kidney lodge where the heminephrectomy was performed. Based on preoperative retrograde pyelography, no correlation of the current collection with the lower pole pelvicalyceal system was observed. It was drained laparoscopically.

**Table 2:** Surgical indication, pathological diagnosis, and karyotype analysis (n = 98). VUR: vesicoureteral reflux; UVJ: ureterovesical junction; LH: luteinizing hormone; CAH: congenital adrenal hyperplasia; FMF: familial mediterranean fever

Operation		Number of operations	Number of patients	Indication	Pathological diagnosis	Karyotype
Ectopic testis exploration/Orchiopexy		14/17 (14.2%/17.3%)	31 (31.6%)	Undescended testicle, non-palpable testicle	-	-
Pyeloplasty		16 (16.3%)	16 (16.3%)	Hydronephrosis, ureteropelvic junction stenosis	-	-
Ovarian pathologies	Ovarian cyst/Tubal cyst	21 (21.4%)	21 (21.4%)	Acute abdomen, abdominal pain	Ovarian cyst/Tubal cyst	-
	Ovarian torsion/Tubal torsion	5/2 (5.1%/2%)		Acute abdomen, abdominal pain		-
	Ovarian mass excision	5 (5.1%)		Abdominal pain, intra-abdominal mass	Dysgerminoma (n = 1). Mature cystic teratoma (n = 3). Serous cystadenoma (n = 1)	-
Partial nephrectomy/Total nephrectomy		6/2 (6.1%/2%)	8 (8.1%)	Duplex collecting system (n = 6), nonfunctional renal (n = 1), renal mass (n = 1)	Nonfunctional renal/Adrenocortical tumor	-
Ureteroneocystostomy		6 (6.1%)	6 (6.1%)	VUR (n = 5), UVJ stenosis (n = 1)	-	-
Bladder diverticulum excision		3 (3%)	1 (1%)	Mesane diverticulum (n = 3)	-	-
Diagnostic		5 (5.1%)	5 (5.1%)	Ductus agenesis, epididim agenesis (n = 1), vaginal atresia (n = 1), intra-abdominal mass (n = 1), pelvic rhabdomyosarcoma (n = 1), LH receptor mutation (n = 1)	-	-
Oophoropexy		2 (2%)	2 (2%)	Ewing sarcoma, bladder rhabdomyosarcoma, radiotherapy	-	-
Gonadectomy		2 (2%)	2 (2%)	Atrophic dysgenetic gonads	Atrophic testicle tissue	46 XY, 46 XY
Bilateral oophorectomy + hysterectomy		1 (1%)	1 (1%)	CAH	-	46 XX
Vaginoplasty		2 (2%)	2 (2%)	Proximal vaginal atresia	-	46 XX
Renal cyst excision		1 (1%)	1 (1%)	Renal cyst	Simple cyst	-
Proximal ureter stone removal		1 (1%)	1 (1%)	Proximal ureter stone	-	-
Lymph node excision		1 (1%)	1 (1%)	FMF, lymph node positivity, elevated sedimentation	Lymphoid hyperplasia	-

A 10-year-old female underwent a simple nephrectomy for high-grade VUR with a left nonfunctional kidney (1.3%). The other case was of a three-year-old boy who was hospitalized due to an intra-abdominal mass. During laparoscopic exploration, the kidney borders could not be clearly distinguished from the kidney parenchyma. A frozen-section material was sent which was compatible with a high-grade renal carcinoma. A left radical nephrectomy was performed. The pathological diagnosis was reported as an adrenocortical tumor.

Of the six patients who underwent laparoscopic UNC, five patients had right VUR, two patients had congenital bladder diverticulum with VUR, and one patient had right ureterovesical junction stenosis. Three of the five patients who underwent UNC had previously received subureteric injections for the treatment of VUR. As the other two patients had bladder diverticula with VUR, subureteric injection was not performed. Two of the patients had grade 4 reflux, two had grade 3 reflux, and one had grade 2 reflux, all of which were on the right side. A ureteral catheter was placed in all patients under cystoscopy guidance to facilitate dissection. All patients who underwent UNC due to VUR underwent a procedure following the extravesical Lich-Gregoir technique. For the patient who ureterovesical stenosis, the modified Lich-Gregoir technique was applied. Bladder diverticulum location was right lateral in two cases and right posterolateral in one case. Diverticulum size was measured as 23, 28, and 30 mm from the smallest measurement on ultrasonography, respectively. Bladder diverticula repair was performed with absorbable sutures.

A 15-year-old male patient was admitted with a kidney cyst measuring 60 × 80 mm in size observed in the upper zone of the left kidney. Therefore, the patient underwent laparoscopic cyst removal. The pathological diagnosis was a simple cortical cyst.

Ureterorenoscopy for a ureteral stone was performed on a nine-year-old male patient with proximal ureteric stone, grade 3 hydronephrosis, and side pain. An obstructed ureteral lumen was observed in the proximal ureter section due to a stone impacting the lumen and mucosa. After laparoscopic exploration, the proximal and distal ureteral parts of the area where the stone was located were clamped with a vascular loop. Then, the stone was removed through a 2 cm vertical ureterotomy.

A two-year-old boy who was operated on for the left undescended testicle, during laparoscopic exploration, rudimentary uterine tissue and Müllerian duct residue were observed and anti-Müllerian hormone deficiency was detected. The testicles were lowered into the scrotum and the rudimentary uterine tissue was excised. Orchidopexy was performed with a two-stage Fowler-Stephens approach in seven patients, while the testicle could be lowered into the scrotum in a single session in 10 patients. In five patients, intra-abdominal testicles and testicles in the inguinal region were not found, and vanishing testicles were detected. In nine patients, the testicle was in the inguinal canal and orchidopexy was completed via the inguinal approach.

All of our patients were followed up jointly with pediatric endocrinology and evaluated in the gender anomalies council and surgical decisions were made.

A patient diagnosed with congenital adrenal hyperplasia with 17 hydroxylase deficiency had a history of bilateral inguinal hernia. Laparoscopic gonadectomy for the atrophic gonads located in the abdomen was performed. The other patient was followed due to luteinizing hormone receptor insensitivity, with karyotype 46 XY and bilateral inguinal atrophic gonads. This patient underwent inguinal gonadectomy combined with diagnostic laparoscopy. One patient, a phenotypic male, due to 21 OH deficiency was due to frequent abdominal pain on the left ovary cyst (6 cm in size). Due to the long-term consequences of suppressing puberty for a longer period and the development of breast tissue, it was evaluated at the council of gender anomalies and a decision was made to develop in a male direction. Laparoscopically, salpingo-oophorectomy and hysterectomy were performed. A 14-year-old and 16-year-old girl presented with primary amenorrhea and hematocolpos. Pelvic ultrasonography and magnetic resonance imaging revealed bilateral ovaries and uterus. In our procedure, the uterus was preserved. The vaginal cavity was determined from the perineum with the help of a bougie. The direction and angle of the cervix and the uterine body were observed, verified, and released with the help of a laparoscope. An incision was made in the cervix to connect the uterine cavity, the endometrial content was aspirated, and a vaginoplasty was created.

Of six patients who presented with acute abdomen, ovarian torsion was detected in five, and tubal torsion was detected in one. In the patient who underwent tubal detorsion with laparoscopy one year ago, torsion developed again in the postoperative third month and was detorsioned again. As there was recurrent tubal torsion, the tuba was fixed to the anterior-lateral wall of the baton. This female patient had been fixed with a tubal non-absorbable suture in her previous surgery. Similarly, a 16-year-old girl presented with an acute abdomen and was diagnosed with a left tubal cyst and tubal torsion. Laparoscopic tubal detorsion and cyst excision were performed. Ovarian cysts were detected in eight patients. Laparoscopic ovarian-sparing cyst excision was performed.

In five female patients, an intra-abdominal mass was detected in the right ovary of four patients and the left ovary of one patient. Mass excision was performed laparoscopically. The pathological diagnoses of the patients are presented in Table 2.

Ovarian-preserving laparoscopic oophoropexy was performed in two patients who were planned for radiotherapy, one patient being followed due to Ewing sarcoma and the other patient being followed due to bladder rhabdomyosarcoma. Both ovaries were fixed to the anterior abdominal wall from the pelvis to the superior.

A 15-year-old girl diagnosed with familial Mediterranean fever had enlarged lymph nodes and her sedimentation level had increased for an unexplained reason. The left parahilar, paraortic lymph node was removed laparoscopically.

The mean duration of operation was 68 (range = 45 to 246) minutes. No intraoperative bleeding occurred and there was no need for conversion to open surgery in any of the patients. Paracetamol was given to all patients for analgesia. The median follow-up time was 13 months.

## Discussion

The use of laparoscopic surgery in pediatric urology continues to develop and expand in recent years. Laparoscopic surgery is a technique with a long learning curve.

A study reported the first series of pediatric transperitoneal laparoscopic dismembered pyeloplasty in 18 children between the ages of three months and 15 years [3]. Multiple series have demonstrated high success rates (92-100%) and low perioperative morbidity. We have gained some insights from our five years of clinical experience. The main ones are that the port entry locations are not standard because the patients are in different age groups with varying weights and heights. We need to choose ports and hand tools according to the patient’s height and weight. In addition, the device quality must be good in terms of display. We think that it is useful to place a suspension suture for traction and to prevent the distal part of the ureter from getting lost among the tissues after the ureter is cut. Some studies support this idea [4]. It is known that laparoscopic surgery causes more physical and mental fatigue than open surgery. Additionally, deterioration in image quality may have a negative impact on the surgeon’s concentration. To ensure better image quality during pelvis excision and anastomosis, fluid restriction was done in consultation with the anesthesia team. In this way, the surgical area is prevented from coming into contact with urine, and deterioration of image quality is prevented. We would also like to suggest that a 4 F double J stent can be easily placed percutaneously through a 14 F branule during the perioperative period for patients who cannot be placed with a retrograde double J stent. Technically, suggestions have been made in the literature to assist in the application of laparoscopy. It is important to position the two surgical ports horizontally to facilitate ergonomic suturing. It may be advisable to leave a small part of the pelvis attached and not separate it completely. This area can be removed once the anastomosis is almost complete. Placing a fixed suture from the ureteropelvic junction to the psoas muscle may assist in visualization of the pelvis and ureter for suturing. When the lower pole crosses the vessels, the pelvis and ureter are separated, and the pelvis must be brought in front of the vessels before the anastomosis begins. It is recommended not to mobilize the ureter more than necessary [5].

In cases with a dual system, the upper pole is sometimes nonfunctional and dilated, which can cause frequent urinary tract infections. As this will affect the entire urinary system, a heminephrectomy is on the agenda. In such cases, laparoscopic surgery can be performed effectively and successfully. However, one of our cases had abdominal pain from time to time even though he had undergone a partial nephrectomy at an external center. In the examination, a cystic lesion was detected in the upper pole of the kidney. The patient underwent laparoscopic cyst excision. During the exploration, it was seen that the upper pole ureter was intact and was not related to the lower pole system. Upper pole partial nephroureterectomy was performed again. In light of this case, it is important to demonstrate the anatomy before nephrectomy. It is crucial to perform retrograde pyelography and visualize both poles of the pelvicalyceal system to clarify the anatomy before partial nephrectomy. We recommend preoperative retrograde ureteral catheterization to prevent ureter and pelvicalyceal system injuries. In addition, in cases where laparoscopic partial nephrectomy will be performed, residual tissue should not be left behind as much as possible and excision can be performed safely with laparoscopic vessel sealers. We think that this suggestion will be effective in eliminating complications.

In one of our patients who underwent total nephrectomy, the diagnosis of a mass originating from the left kidney and the simultaneous evaluation of the opposite kidney were the conveniences provided by laparoscopy. The mass, which did not invade surrounding tissues, was removed by total nephrectomy. The mass excision of the patient, whose pathology indicated an adrenocortical tumor, which is very rare in the pediatric age group [6], was performed safely laparoscopically. The patient was followed postoperatively for three years without any problems. The case was presented previously as a case report [7].

Laparoscopic extravesical and pneumovesicoscopic UNC are the methods used in today’s pediatric urology practice. In the pediatric age group, UNC is most commonly performed due to VUR and lower-end ureteral stenosis. UNC is commonly performed laparoscopically via the pneumovesicoscopic method. However, the issue of extravesical UNC remains unclear. Although not yet as promising as the open method, the first results of pneumovesicoscopic reimplantation with the Politano-Leadbetter technique are reported to be promising [8]. Extravesical UNC was performed in five of our patients due to VUR and one due to ureteral lower-end stenosis. We created a dissection plan by preoperative cystoscopy and placement of a retrograde ureteral catheter for our patients with unilateral VUR and loss of kidney function. We would like to emphasize that caution should be exercised in terms of injury to the iliac vessels and ductus deferens in male patients. Extravesical UNC can be performed successfully by laparoscopic method, particularly in cases with diverticula. One of the factors that should be taken into consideration in this application is the placement of a ureteral stent in the first applied cases for ease of finding the ureter. We recommend that the sutures used during detrusor repair be separated to ensure their durability and prevent diverticulum recurrence.

Literature regarding surgical management of pediatric renal cysts is limited, as pediatric cases are often grouped with adults. Conservative monitoring is recommended for uncomplicated asymptomatic cysts [9]. The onset of hematuria, pain, or hypertension requires more invasive treatment. In our case, laparoscopic cyst excision was performed successfully and safely, as flank pain and an increase in cyst size (8 cm) were detected.

Laparoscopic ureterolithotomy for large proximal ureteral stones appears to have a higher stone-free rate and lower postoperative ureteral stenosis rate compared to ureteroscopic lithotripsy [10]. We performed laparoscopic ureterolithotomy on our patients by clamping the proximal and distal stones with a vascular sling to prevent the stones from moving. The ureterotomy was closed using 6-0 Vicryl intermittent sutures. No ureteral stenosis was observed in the patient who was followed up without a ureteral stent. Laparoscopic ureterolithotomy is safe when performed by well-trained laparoscopic surgeons and has few major perioperative complications [11]. According to our experience and literature, we recommend laparoscopic ureterolithotomy for patients with large impacted proximal ureteral stones.

The first publication on the use of laparoscopy in the diagnosis of nonpalpable testicles (NPTs) was reported by Cortesi et al. [12] in 1976. In our clinic, as suggested by Dhannani et al. [13], we performed primary orchidopexy in cases where the distance was between 2 and 4 cm and if the length of the spermatic vessels reached the inguinal ring on the opposite side of the testicle. We generally performed two-stage orchidopexy when the distance of the testicle to the internal ring was 4 cm or more (highly located intra-abdominal testicles). During the one-year follow-up, testicular atrophy was observed in a patient who underwent two-stage orchidopexy. It has been reported that laparoscopic one-stage orchiopexy gives good results in low-lying intra-abdominal testicles, while the results of the two-stage Fowler-Stephens technique are also good in high-lying intra-abdominal testicles [14]. Some studies recommend the inguinal approach first in unilateral NPTs with the view that laparoscopy should be performed only for bilateral cases [15,16]. In our series, rudimentary uterine tissue was observed in the abdomen of a patient with unilateral NPT, and anti-Müllerian hormone deficiency was detected as a result of endocrinological investigation. In our clinic, we performed laparoscopic exploration for all patients with unilateral NPT. We support our approach with the findings we found in this patient. In addition, we think that patients with unilateral NPTs and no other abnormal genital examination should be evaluated by the pediatric endocrinology clinic before the procedure.

Laparoscopy is a useful method in the diagnosis and treatment of sexual anomalies due to its minimal invasiveness and positive cosmetic results. Gonadectomy is indicated in several intersex disorders involving the Y chromosome to reduce the risk of associated cancer [17]. Two patients in our study underwent laparoscopic gonadectomy without any problems. Postoperative complications were reported in only one series and included one umbilical port infection and one pelvic abscess, both of which were treated with antibiotic therapy [18]. Our patients continue to be followed up without any problems in the second postoperative year. We think that laparoscopic gonadectomy should be considered the preferred treatment method in children and adolescents with these rare conditions. The advantages of the minimally invasive approach include lower morbidity, faster postoperative recovery, and elimination of the risk of malignancy of gonadal origin.

Proximal vaginal atresia is a rare condition worldwide. The majority of reported cases involve an absent uterus [19]. In patients with a functional uterus, fertility-preserving surgery should be the primary choice. Based on our experience, we can conclude that laparoscopy-assisted vaginoplasty is a promising method to achieve ideal results for fertility and sexual life.

Laparoscopic detorsion is the current treatment method in the treatment of ovarian torsion [20]. Laparoscopic ovarian torsion was performed without any problems in five patients in our series. One of the two patients with isolated tubal torsion also had a tubal cyst. The cyst was excised and detorsioned. No pathology was detected in the ovaries of either patient. In the literature, isolated tubal torsion is reported in a limited number of case reports in the pediatric age group [21,22]. One patient developed recurrent tubal torsion and the tube was fixed to the anterior abdominal wall. In the patient who developed relapse, it was observed that torsion developed after COVID-19 infection. We do not have such a theory, but it was remarkable that it occurred simultaneously with the COVID-19 infection. In the second postoperative year, follow-up continues without any problems.

Ovarian masses can be removed laparoscopically. Ovarian teratomas are benign or malignant neoplasms originating from the primordial germ cells of the ovary. Overall. 18% of ovarian masses are mature teratomas and have a benign character and a distinct cystic structure [23]. Oophorectomy has been used for years in cases such as large cysts, suspicion of latent germ cell malignancy, presence of torsion, decreased viability of the remaining ovarian tissue, and content shedding [24].

The pathology results of patients who underwent surgery due to ovarian mass were mature cystic teratoma (n = 3), serous cystadenoma (n = 1), and dysgerminoma (n = 1). Malignant ovarian tumors in childhood are extremely rare. Dysgerminoma is the most common malignant ovarian tumor, accounting for 26.8% of pediatric malignant ovarian tumors [25].

Epithelial tumors are rare in the prepubertal age group. Tumors of surface epithelial cell origin are less common, with the most common being serous cystadenoma [26]. Although serous cyst adenoma of the ovary is rare in adolescents, it is important to keep epithelial tumors in mind in the differential diagnosis of ovarian masses and torsions and to ensure close follow-up due to recurrences after surgery. Our patient, a 16-year-old girl, underwent total excision of a 15 cm mass originating from the left ovary. The pathology result was reported as serous cystadenoma. We think that in cases where a malignant mass is suspected, one should not be conservative in widening the incision through which the mass is removed to avoid perforation while removing the mass.

Pelvic irradiation treatment is required for some types of cancer in the pediatric age group. In such cases, gonad-protective treatment comes to the fore to prevent the gonads from being affected by the radiation. In such a situation, the first thing that comes to the families’ minds is the treatment of the current disease. However, this can be a problem when productivity comes to the fore in the long run. Hence, by taking ovarian-protective measures and informing the families, psychological, sociological, and physiological problems in the future can be prevented. Radiation plates for ovarian-protective methods may not be effective in the pediatric age group. The ovary may need to be removed from the irradiation area and moved to an extrapelvic intra-abdominal area. This procedure can be performed by laparotomy or laparoscopically. In our two patients who were followed up due to bladder rhabdomyosarcoma and Ewing sarcoma, radiotherapy was required during the treatment process. Hence, the ovaries were released from their ligaments by laparoscopic method, fixed under the spleen and liver, and removed from the pelvic region. We agree with some studies that this issue should not be left aside during the diagnosis and treatment process in today’s conditions where successful fertility preservation methods are available [27].

Lymph node excision performed on a patient with an unexplained high sedimentation rate and multiple bilateral paraaortic parahilar lymph nodes can be used as a safe method for all kinds of diagnostic exploration, as in our patient, with laparoscopic surgical procedure.

Our study has several limitations. We did not compare our study cohort with a control group of children undergoing open surgery. Postoperative pain management was not standardized, and therefore could not be reported. Furthermore, the diversity in patient diagnoses and procedures made it challenging to draw clear conclusions and implement standardization.

## Conclusions

The laparoscopic method should be performed by surgeons experienced in advanced surgeries in pediatric urology. It is crucial to consider the size difference of pediatric patients in preparation for laparoscopic surgery to minimize technical and ergonomic problems. We believe that each surgery has its own specific tricks and that these should be a part of laparoscopy training. Developing and sharing this information will be very useful for pediatric urologists.
